# Commentary on: Use of Himplant® for correction of residual deformity following prior treatment of Peyronie’s disease: a case report

**DOI:** 10.1038/s41443-024-00932-4

**Published:** 2024-07-05

**Authors:** Wai Gin Lee, Karl H. Pang

**Affiliations:** 1 St Peter’s Andrology, London, UK; 2 University College London Hospitals NHS Foundation Trust, London, UK; 3 The Division of Surgery and Interventional Sciences, University College London, London, UK

**Keywords:** Surgery, Sexual dysfunction

Interest in silicone sleeve devices is increasing among patients and clinicians, with the number of centres offering this device rising across the United States (US) in recent years [1]. The devices are FDA approved to correct soft tissue penile deformities [2] and for penile enhancement [3]. The original device (Penuma®, International Medical Devices, Los Angeles, CA, USA)) was constructed with 2 Dacron (DuPont, Wilmington, DE, USA) mesh layers encased within medical-grade silicone [4]. Tissue ingrowth was encouraged by suturing a double layer of polypropylene mesh (Parietex; Covidien, Dublin, Ireland) folded over the distal end of the implant. The device was modified in 2023 by integrating the external mesh into the design of the device and it was rebranded the Himplant® device. No reports have been published from outside of the US.

This case series by Levine et al reports use of the updated Himplant® device. Importantly, it also reports on the expanding use of silicone sleeve implants for the correction of underlying penile pathology. In the present series, 3 men with residual penile deformity following treatment of Peyronie’s disease (surgical plication and/or collagenase injections) were included [5]. Patient 1 had a residual narrowing in the penile shaft and loss of penile volume and girth; while patient 2 had a proximal shaft narrowing and a right lateral indentation (without hinging) and a residual dorso-left lateral curvature of up to 40 degrees. Patient 3 reported midshaft narrowing and a residual dorsal curvature of 30 degrees. Men with penile instability or buckling (hinge-effect) were judged poor candidates and therefore excluded from receiving the device.

Functional outcomes and satisfaction were measured by IIEF questionnaire and a 3-item non-validated 5-scale questionnaire on overall satisfaction and satisfaction with penile girth enhancement and correction of curvature. At follow-up of 14-19 months, all patients were sexually active and were engaging in penetrative intercourse. Penile deformity was completely masked in all 3 patients and the flaccid dorsal length increased by 1.4-1.6 cm (mean, 1.5 cm) and the flaccid midshaft girth increased by 1.5-2.3 cm (mean, 2 cm). All patients were “highly” or “very highly” satisfied with the implant, enhanced penile girth and penile deformity correction. Seroma occurred in 2 out of the 3 patients and were managed conservatively by aspiration in clinic. No other complications were reported at 19 months follow-up.

This study is a welcome and much needed addition to the literature on silicone sleeve device despite the limitations of the study design and short follow-up. Data from the evaluation of the Penuma® device suggested that at longer follow-up, the “high” and “very high” satisfaction rates decreased from 100% [4] to 57-81% [1, 6]. In addition, late complications requiring explant (infection, breakage, haematoma or patient request) were reported in 3-10% of men [1, 6, 7].

Nevertheless, this study builds on the recent (and first) evaluation of the Himplant® device in 4 patients who experienced refractory erectile dysfunction and penile retraction following radical prostatectomy [8]. A mean increase in dorsal flaccid length and midshaft girth of 4.4 cm and 3.6 cm respectively was reported. All patients noted improved erectile function without the use of phosphodiesterase-5 inhibitors. No complications or further interventions were reported up to 55 months postoperatively. These two studies join a recent study in men with retractile/buried penis [9] where the device was not inserted for purely cosmetic reasons.

Significant deficiencies remain in the published literature on silicone sleeve devices despite the present study. Firstly, the Himplant® and the Penuma® devices have not been compared hence the overall benefits of the newer device are not known. In addition, outcomes and patient satisfaction following silicone sleeve implantation have not been compared to other surgical and non-surgical techniques. Extratunical grafting using cadaveric fascia lata graft (Tutoplast Suspend®) have shown satisfactory resolution of penile indentation from Peyronie’s disease at an average follow-up of 21 months [10]. Non-surgical treatments including the injection of hyaluronic acid and polylactic acid fillers [11] and autologous fat [12] may result in penile girth increase of up to 2 cm. However, complications from these injections include bleeding, infection, and nodule formation [11–13], and excision of the injected material may be required [14].

In summary, larger well-characterised series with longer-term and complete follow-up are required to assess the true safety, durability and efficacy of Himplant®. In addition, there are no validated questionnaires for the Himplant®, therefore, standardised reporting is required to enable non-heterogenous analysis in the future. There is a need for more robust studies comparing the device to other therapeutic options. Lastly, clinicians offering these devices are encouraged to consider the recommendations of the recent Sexual Medicine Society of North America (SNSNA) position statement on cosmetic penile enhancement procedures [15].
